# PRR11 Is a Prognostic Marker and Potential Oncogene in Patients with Gastric Cancer

**DOI:** 10.1371/journal.pone.0128943

**Published:** 2015-08-07

**Authors:** Zongchang Song, Wenying Liu, Yu Xiao, Minghui Zhang, Yan Luo, Weiwei Yuan, Yu Xu, Guanzhen Yu, Yide Hu

**Affiliations:** 1 The Third Department of Oncology, The Second Affiliated Hospital of The Third Military Medical University, Chongqing, 400037, China; 2 Department of Medical Oncology, Shanghai East Hospital, Shanghai, 200120, China; 3 Department of Medical Oncology, Changzheng Hospital, Shanghai, 200070, China; The University of Texas MD Anderson Cancer Center, UNITED STATES

## Abstract

PRR11 is a potential candidate oncogene that has been implicated in the pathogenesis of lung cancer, however the role of PRR11 in gastric cancer is currently unclear. In the present study, we investigated the role of PRR11 in gastric cancer by evaluating its expression status in samples from a cohort of 216 patients with gastric cancer. PRR11 was found to be overexpressed in 107 (49.5%) patients by immunohistochemistry of tissue microarrays generated using the patient samples. Furthermore, PRR11 overexpression was found to correlate significantly with clinicopathologic features such as tumor invasion, tumor differentiation, and disease stage. Survival analysis of the cohort revealed that PRR11 is an independent prognostic factor for gastric cancer patients. PRR11 was stably silenced in a gastric carcinoma cell line using an shRNA-based approach, and treated cells showed decreased cellular proliferation and colony formation in vitro and cell growth in vivo, companied by decreased expression of CTHRC1 and increased expression of LXN, proteins involved in tumor progression. Evaluation of human gastric cancer samples demonstrated that PRR11 expression was also associated with increased CTHRC1 and decreased LXN expression. These data indicate that PRR11 may be widely activated in human gastric cancer and are consistent with the hypothesis that PRR11 functions as an oncogene in the development and progression of gastric cancer.

## Introduction

Gastric cancer (GC) is the fourth most common malignancy worldwide with an estimated incidence of one million new cases in 2008, accounting for 8% of new cancers. The mortality rate associated with GC is also staggering, with 0.73 million deaths accounting for 10% of total cancer-related deaths estimated for 2008. Of note, approximately 70% of new GC cases and GC-related deaths occur in developing countries [1]. Although there have been important medical advances in the diagnosis and treatment of GC over the past few decades, this disease remains the second most common cause of cancer-related mortality in the world partially due to the fact that it is commonly detected in patients with late stage disease, abrogating successful curative surgery for many patients[1, 2].

While the incidence of GC rates has decreased substantially in North America and in most Northern and Western Europe, the disease is still prevalent in Eastern Europe, Russia, Central and South America, and East Asia [3]. Currently there are several novel tissue-based prognostic and therapeutic markers for gastric cancer. These include human epidermal growth factor receptor-2 (HER2) [4, 5], vascular endothelial growth factor receptor 2 (VEGFR-2) [6], excision repair cross-complementation group 1(ERCC1) [7], B-cell lymphoma-2(Bcl-2) and Ki-67 [8]. However, most of these markers are not routinely were used in clinical practice because they do not accurately and efficiently predict outcome or therapeutic efficiency. There is currently a great clinical demand for novel molecular markers that can improve detection, diagnosis, and prognostication of gastric cancer and eliminate the need for costly and inefficient endoscopic screening methods. In 2013, proline-rich protein 11 (PRR11) was identified as a novel gene and functionally characterized by Ji Ying et al. who discovered that PRR11 has an important role in both cell cycle progression and tumorigenesis. This protein, due to its oncogenic role, has been indicated as a potential novel target in the diagnosis and treatment of human lung cancer. Through regulating important genes involved in cell cycles and tumorigenesis, PRR11 participates in the initiation and progression of lung cancer [9] and epithelial-to-mesenchymal transition in breast cancer[10].

However, at present, knowledge concerning the role of the PRR11 in gastric cancer has not been previously reported. In this study, we evaluated the PRR11 expression status in a cohort of 216 patients with GC and analyzed the relationship between PRR11 expression and clinicopathological parameters to determine whether PRR11 can predict GC patient prognosis. In addition, silencing of PRR11 in multiple gastric carcinoma cell lines inhibited cellular proliferation rates, cancer cell migration in cell colony formation, and tumor growth in vivo experiment. These findings are important because they are consistent with previous hypotheses that PRR11 may be an important oncogenic factor in a variety of cancer.

## Methods

### Cohort selection and tissue acquisition

The cohort consisted of 216 patients with GC that received surgical resections at Changhai Hospital in Shanghai, People’s Republic of China, from 2001 to 2005. Patients’ follow-up was received in clinical reports until March 2010, and each patients was confirmed to have sufficient amount of GC store for constructing a tissue microarray (TMA) Among the patient information collected were characteristics including age, gender, tumor size, T stage, N stage, M stage, and tumor differentiation (Table 1). All tissue specimens were obtained after patients provided written informed consent. The experimental design was approved by the Changhai Hospital Institutional Review Board prior to the study.

**Table 1:** Correlation between overexpression of PRR11 and clinicopathological of gastric cancer.

			PRR11	
Variables		N	Positive (%)	*P*
Age				
	≤60y	71	31(43.7)	0.227
	>60y	145	76(52.4)	
Gender				
	Male	149	74(49.7)	0.955
	Female	67	33(49.3)	
Tumor size				
	≤6cm	180	84(46.7)	0.059
	>6cm	36	23(63.9)	
T stage				
	T1/2	78	26(33.3)	<0.001
	T3/4	138	81(58.7)	
N stage				
	N0	86	36(41.9)	0.066
	N1-3	130	71(54.6)	
Differentiation				
	Well/moderate	143	59(41.3)	0.001
	Poorly/undifferentiated	73	48(65.8)	
TNM stage				
	I/II	101	41(40.6)	0.014
	III/IV	115	66(57.4)	

### TMA and Immunohistochemistry and Scoring

Tissue microarrays were constructed in a manner previously [11]. Briefly, H&E-stained slides from all patients were reviewed and identified by two anatomic pathologists and the representative tumor-containing portions were pre-marked in the paraffin blocks. Tissue cylinders with a diameter of 1.5 mm were punched from the marked areas of each block and incorporated into a recipient paraffin block. Sections 4-μm thick were placed on slides coated with 3-aminopropyltriethoxysilane. Paraffin sections were deparaffinized in xylene and rehydrated using decreasing concentrations of ethanol (100%, 95%, and 85%, 5 min each). Antigen-retrieval was accomplished by microwave irradiation for 3 min in pH 6.0 citric buffer and cooled at room temperature for 120 min. Endogenous peroxidase activity was blocked by incubation of the slides in 3% H2O2/phosphate-buffered saline, and nonspecific binding sites were blocked using goat serum. Primary antibodies were diluted as follows: anti-PRR11 (1:100; HPA023923, Sigma-Aldrich, St. Louis, MO, USA), anti-LXN (Latexin) (1:100; GT39981-16; Sigma-Aldrich); anti-CTHRC1 (1:100; 11165-1G12; Sigma-Aldrich). An IHC staining S-P kit (KIT-9710; MAIXIN Biology Corporation, Fuzhou, China) was used to visualize antibody binding on the slides. Counterstaining was performed with hematoxylin. The IHC staining of PRR11, LXN, and CTHRC1 in these specimens was evaluated using an Olympus CX31 microscope (Olympus, Center Valley, PA). Tumors that demonstrated >10% staining for PRR11 were considered as having positive expression status

### Cell lines and culture conditions

Protein expression was detected using the five GC cell lines SGC7901, MKN45, MKN28, HGC27, and MGC803, which were purchased from Cell Bank, Chinese Academy of Sciences. GC cell lines were maintained in Dulbecco's modified Eagle's medium (DMEM) (Hi-Clone) plus 10% FBS at 37°C with 5% CO2.

### Knockdown of PRR11 by lentiviral vector-loaded shRNA in GC cells

Packing of lentiviral particle was performed in the following manner. Briefly, lentivirus expression plasmid containing small interference RNA (5’-ACGCAGGCCUUAAGGAGAATT-3’) targeting PRR11 was constructed by GENECHEM Corporation (Shanghai, China) and used for infecting the GC cells in presence of 6 μg/ml polybrene. Infected gastric cancer cells were selected for using puromycin, and knockdown of PRR11 was confirmed by immunoblotting the cell lysates with anti-PRR11 antibody.

### Cell proliferation assay and colony formation assay

Cells with stably-transfected PRR11-shRNA (PRR11-KO) or empty vector (control) were seeded in 96-well plates at a density of 5,000 cells per well. CCK8 assays (Dojindo Kumamoto, Japan) were performed as a measure of proliferation, which was calculated as the ratio of the absorbance at 48h compared with that at 24h.

Colony formation assays were conducted in a similar manner. PRR11-KO and control cells were seeded into 6-well plates, in triplicate, at a density of 500 cells per well. Two weeks after incubation, the colonies were fixed with methanol/acetone (1:1), stained with crystal violet, and counted. Colonies with less than 50 cells per well were considered negative for colony formation.

### Western blot

Total protein was prepared from lysates of SGC7901, MKN45, MKN28, HGC27, and MGC28 gastric carcinoma cell lines. Western blots were performed in the standard manner using a goat polyclonal antibody against human PRR11 (dilution, 1:1000), LXN (dilution, 1:1000), CTHRC1 (dilution, 1:1000) and a horseradish peroxidase-conjugated anti-goat IgG antibody diluted 1:10000 as the secondary antibody. Expression of ß-actin was evaluated to normalize measurements of protein expression. The proteins were visualized using the Amersham enhanced chemiluminescence system according to the manufacturer’s instructions with Image J software.

### Real-Time Quantitative Reverse transcription (qRT)-PCR Assay

Real-time quantitative PCR reaction was performed using SYBR1 Premix Ex TaqTM kit (Takara, Kyoto, Japan) according to the manufacturer’s instructions and conventional PCR assays were performed as previously described[12]. GAPDH was used as an internal standard. The PCR cycle protocol used the following parameters: 95 degree for 1 min, 40 cycles of 15 sec at 95 degree and 31 sec at 60 degree. The fold-expression was calculated using the ΔCt method. Reactions and analysis were performed using the ABI PRISM 7300 PCR and detection system (Applied Biosystems, Carlsbad, CA). The primers used are as follow: PRR11: forward: 5’- CGT ATC TGC CAC CGA GAA CTT-3’, reverse: 5’- GAG ATG GTC TTC AGT GCT TCC T-3’; GAPDH: forward: 5’-TGA CTT CAA CAG CGA CAC CCA-3’, reverse: 5’- CAC CCT GTT GCT GTA GCC AAA-3.

### Animal models

Subcutaneous tumor xenograft models were performed to evaluate the in vivo function alteration of PRR11-knockdown in SGC-7901 cell line. PRR11-KO SGC-7901 cells and controls (1×106 cells in 0.1mL PBS) were injected subcutaneously into the left flank of 4-week-old female Balb/c nude mice (the Animal Center of the Second Military Medical University) (n = 5). Tumor diameter was measured every three days. Two weeks after implantation, all animals were sacrificed. The tumors were collected and tumor volume (mm3) was calculated as V = 0.52 (length×width×depth). This animal experiment was approved by the Animal Ethics Committee of the Second Military Medical University.

### Statistical Analysis

Statistical analyses were performed using SPSS 13.0 software (SPSS, Chicago, IL, USA). The relationships between the expression of PRR11 and clinicopathological characteristics was evaluated using chi-squared distributions. GC patients were stratified according to the expression status of PRR11, LXN, and CTHRC1, and survival analysis were performed using Kaplan-Meier curves. Univariate and multivariate analyses were based on a Cox proportional hazard regression model. The variables showing significance (p<0.05) by univariate analysis were adopted when multivariate Cox proportional hazards analysis was performed. Quantitative variables were analyzed using Student’s t-test. Experimental data were presented as the mean of each condition ± S.D. and p < 0.05 were considered statistically significant.

## Results

### PRR11 expression status correlates with important clinicopathological characteristics of gastric cancer

**Fig 1:**
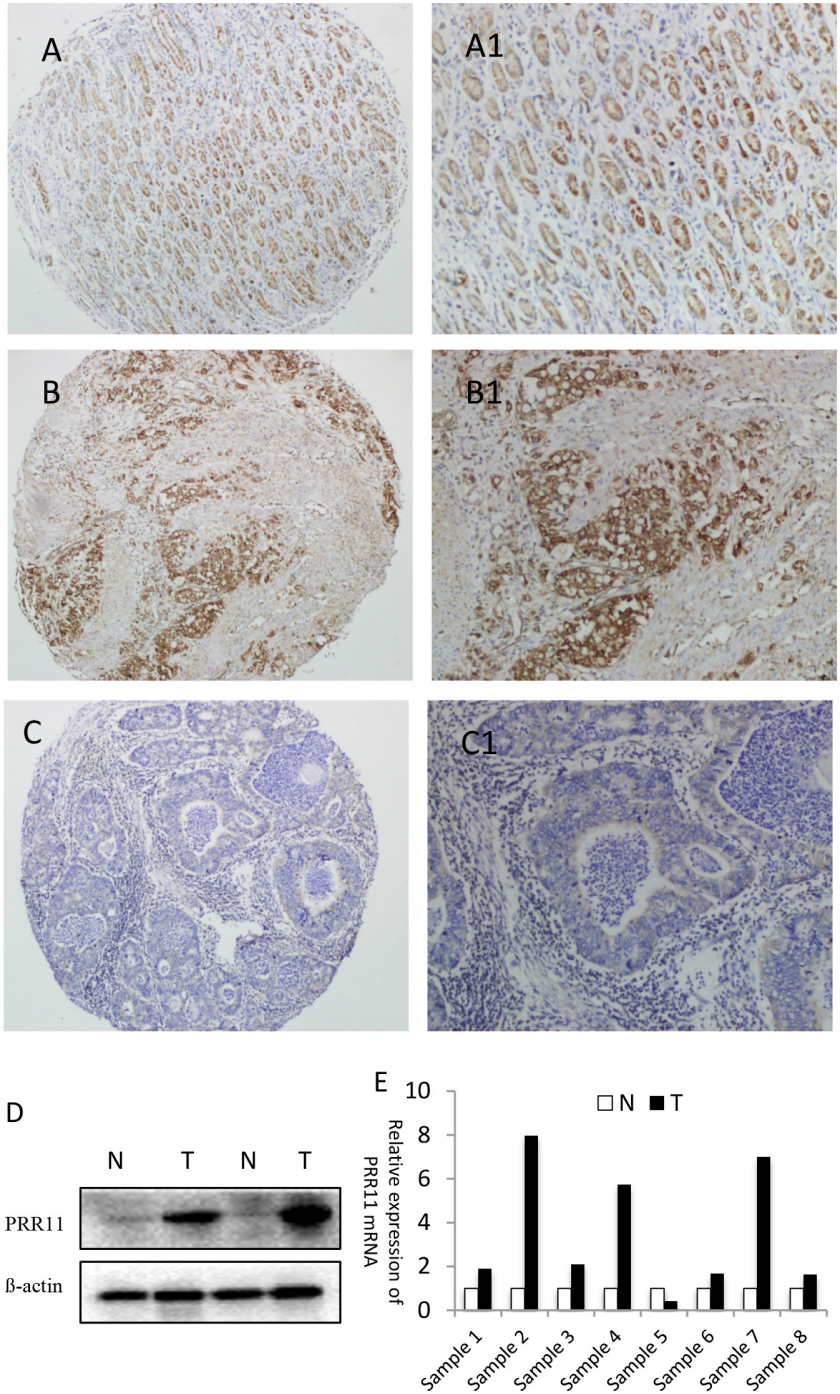
Analysis of PRR11 expression in human gastric cancers and adjacent normal mucosa specimens. (A, A1) Normal gastric mucosa showed weak staining of PRR11; (B, B1) Positive staining of PRR11 in gastric cancer; (C, C1) Negative staining of PRR11 in gastric cancer. A1, B1, C1 are enlargement of tissues from A, B, C, respectively. Original magnification of A, B, C: 40×; Original magnification of A1, B1, C1: 200×. (D) Western blotting revealed increased expression of PRR11 in tumor samples (T) compared with that in noncancerous tissues (N); (E) mRNA levels of PRR11 in gastric cancer tissues (T) and normal tissues (N).

The expression of PRR11 was evaluated by immunohistochemistry in a cohort of 216 patients with gastric carcinoma. Low intensity of cytoplasmic PRR11 immunostaining was visible in the normal epithelial cells (Fig 1A). High intensity of PRR11 immunostaining was observed specifically in gastric cancer cells, and the staining intensity was variable between patients (Fig 1B and 1C). Of these tumor samples, 107 out of 216 (49.5%) had positive PRR11 expression and 109 cases showed PRR11 negative expression (Fig 1C). Western blotting and RT-PCR assay further confirmed that PRR11 protein and mRNA was increased in tumor samples compared with that in the noncancerous tissues (Fig 1D and 1E). Complimenting these results, a relatively high level of PRR11 protein expression level was also detected in five GC cell lines, including MKN45, MKN28, SGC7901, HGC27, and MGC803 (S1 Fig).

PRR11 expression correlated significantly with number of clinicopathological parameters such as T stage, degree of tumor differentiation, and TNM stage (Table 1). PRR11 expression was not found to be associated with age, gender, tumor size, and N stage. These results suggest that high PRR11 protein expression is associated with an aggressive phenotype of gastric cancer.

### PRR11 tumor expression is associated with decreased survival in gastric cancer patients

**Fig 2:**
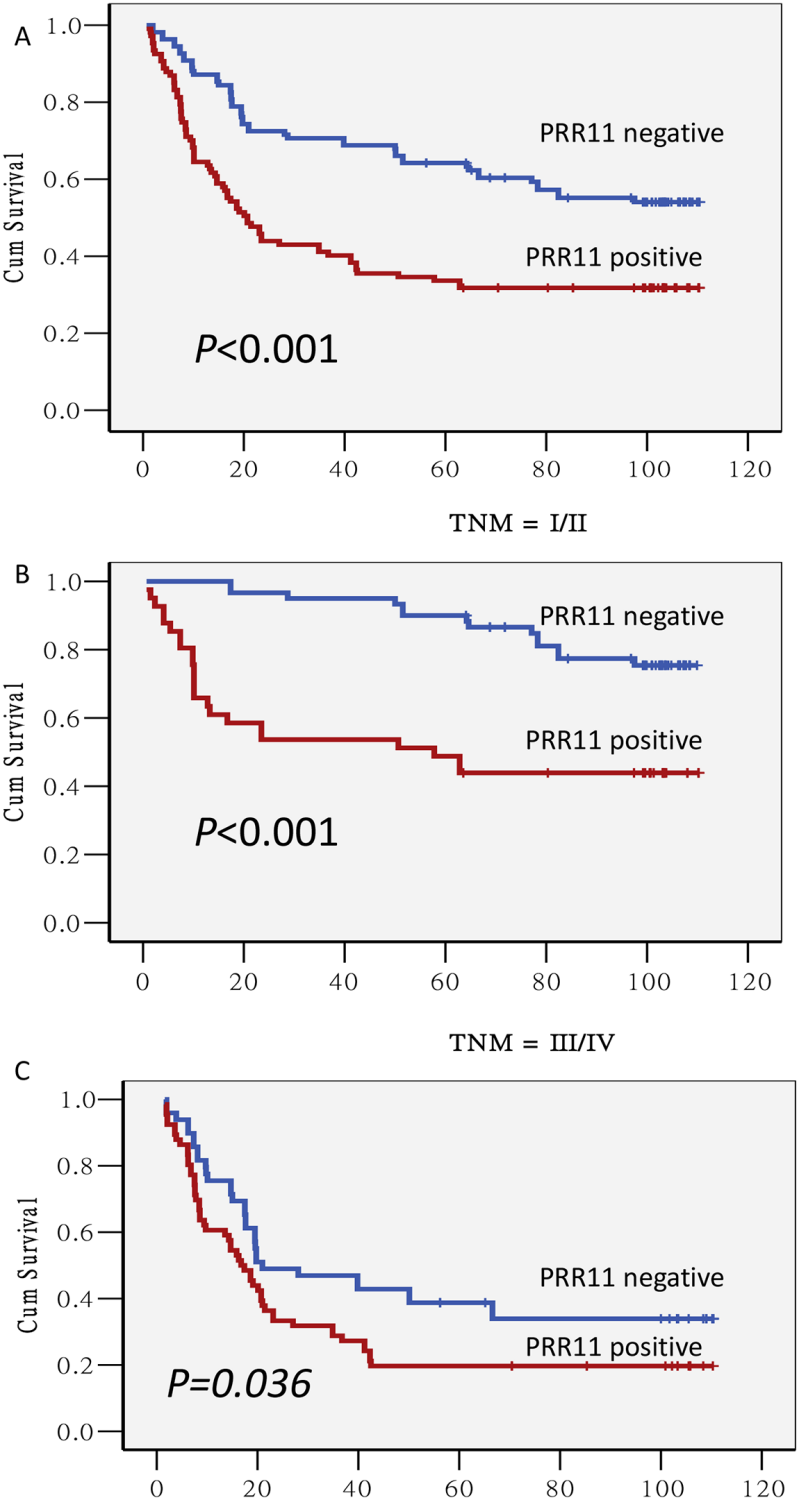
Kaplan-Meier survival curves of gastric carcinoma patients with and without tumor expression of PRR11. (A) Survival was significantly longer in patients with tumor lacking expression of PRR11 (OS, 76.6 month) versus those with positive PRR11 expression status (OS, 46.6 month; P < 0.001). (B) A subgroup analysis of stage I & II patients revealed that patients with a positive PRR11 expression status was associated with a shorter survival duration than patients without PRR11 expression (58.5mon vs. 97.9mon; *P*<0.001). (C) A subgroup analysis of stage III & IV patients demonstrated that positive PRR11 overexpression was associated with a shorter overall survival than patients without PRR11 expression (34.3mon vs. 51.3mon; *P* = 0.036).

Among the 216 patients studied, 122 died during the follow-up period with a median overall survival time of 51.5 months. The mean survival time for patients with negative PRR11 protein expression was 74.5 months, while the mean survival time for patients with positive PRR11 protein expression was 46.4 months (Fig 2A).

Univariate COX regression analyses demonstrated that tumor size, tumor stage, lymph nodes positivity, TNM stage, tumor differentiation and PRR11 expression were significantly correlated with a decreased overall survival (Table 2). Multivariate analysis confirmed that tumor size, tumor differentiation and PRR11 expression were independent prognostic predictor of overall survival of gastric cancer patients (HR = 0.663; 95% CI: 0.406–0.961 for tumor size, HR = 0.545; 95% CI: 0.372–0.798 for PRR11, HR = 0.548; 95% CI: 0.376–0.801 for tumor differentiation).

**Table 2:** Univariate and Multivariate Analysis of Variables Associated with OS in Patients with gastric cancer.

Variable		No.	Mean Survival (months)	*P* (univariate)	*P* (multivariate)	Hazard Ratio	95% CI
Tumor size							
	≤6cm	180	66	<0.001	0.032	0.663	0.406 to 0.961
	>6cm	36	34				
Tumor stage							
	T1/T2	78	85	<0.001	0.183	0.663	0.362 to 1.214
	T3/T4	138	46				
Regional lymph nodes positive							
	No	86	78	<0.001	0.570	1.237	0.594 to 2.578
	Yes	130	49				
TNM stage							
	I/II	101	82	<0.001	0.060	0.453	0.198 to 1.034
	III/IV	115	42				
PRR11							
	Negative	109	77	<0.001	0.002	0.545	0.372 to 0.798
	Positive	107	44				
Differentiation							
	well/moderate	143	71	<0.001	0.002	0.548	0.376 to 0.801
	poor/undifferentiated	73	39				

Patients were divided into two groups, those with TNM stage I/II and those stage III/IV, and the effect of PRR11 status on survival duration was assessed. This subgroup analysis revealed that the outcomes of patients with PRR11 overexpression were worse than those without PRR11 overexpression either in stages I/ II (Fig 2B) or in stage III/IV (Fig 2C).

### PRR11 expression is associated with increased cellular proliferation and cell colony formation in vitro and tumor growth in vivo in SGC7901 gastric carcinoma cells

**Fig 3:**
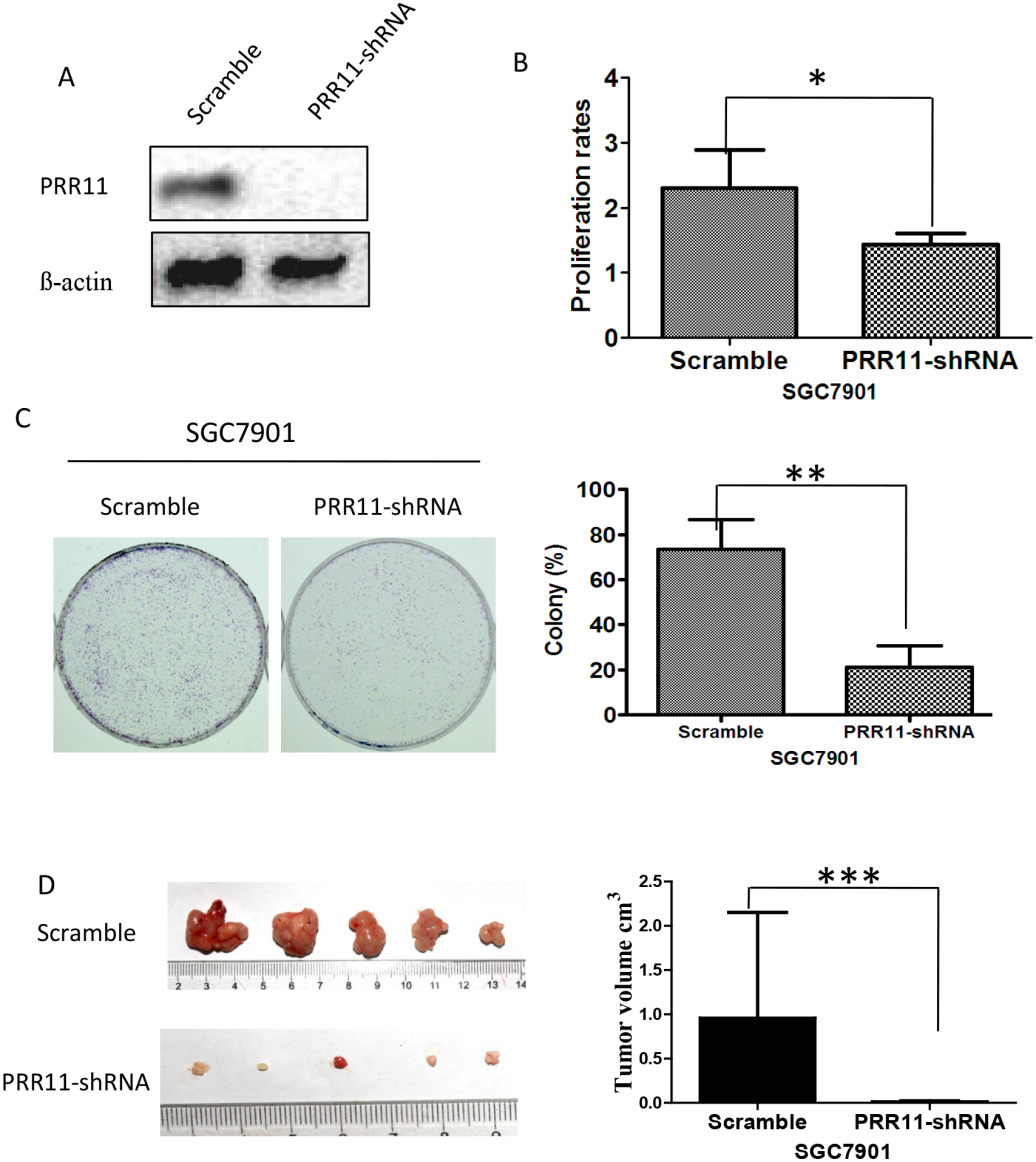
Knock down of PRR11 inhibited cell proliferation and GC cell colony forming ability in the SGC-7901 gastric carcinoma cell line. (A) Western blot analysis of PRR11 expression at 48 hours after transfection of PRR11-shRNA in SGC7901 cells; (B, C) Silencing of PRR11 inhibits cell proliferation (B) and colony forming ability in vitro (C). (D) Silencing of PRR11 inhibited tumor grow in vivo. *P<0.05, **P<0.01, **P<0.01 considered statistically significant compared with control.

PRR11 was stably silenced in a SGC-7901 cell line using lentiviral vector-loaded PRR11 shRNA. Knockdown of PRR11 (PRR11-KO) was confirmed via Western blot analysis, and the effect of PRR11-KO on cellular proliferation and colony formation was evaluated. Significant depletion of PRR11 in the transfected cells was observed (Fig 3A), and down-regulation of PRR11 was associated with a dramatic decrease in both cell proliferation (Fig 3B) and cell colony formation (Fig 3C) in this cell line. Unsurprisingly, PRR11 down-regulation leads to retarded growth of gastric cancer cell lines in vivo (Fig 3D)

### Knockdown of PRR11 results in down-regulation of CTHRC1 and up-regulation of LXN

**Fig 4:**
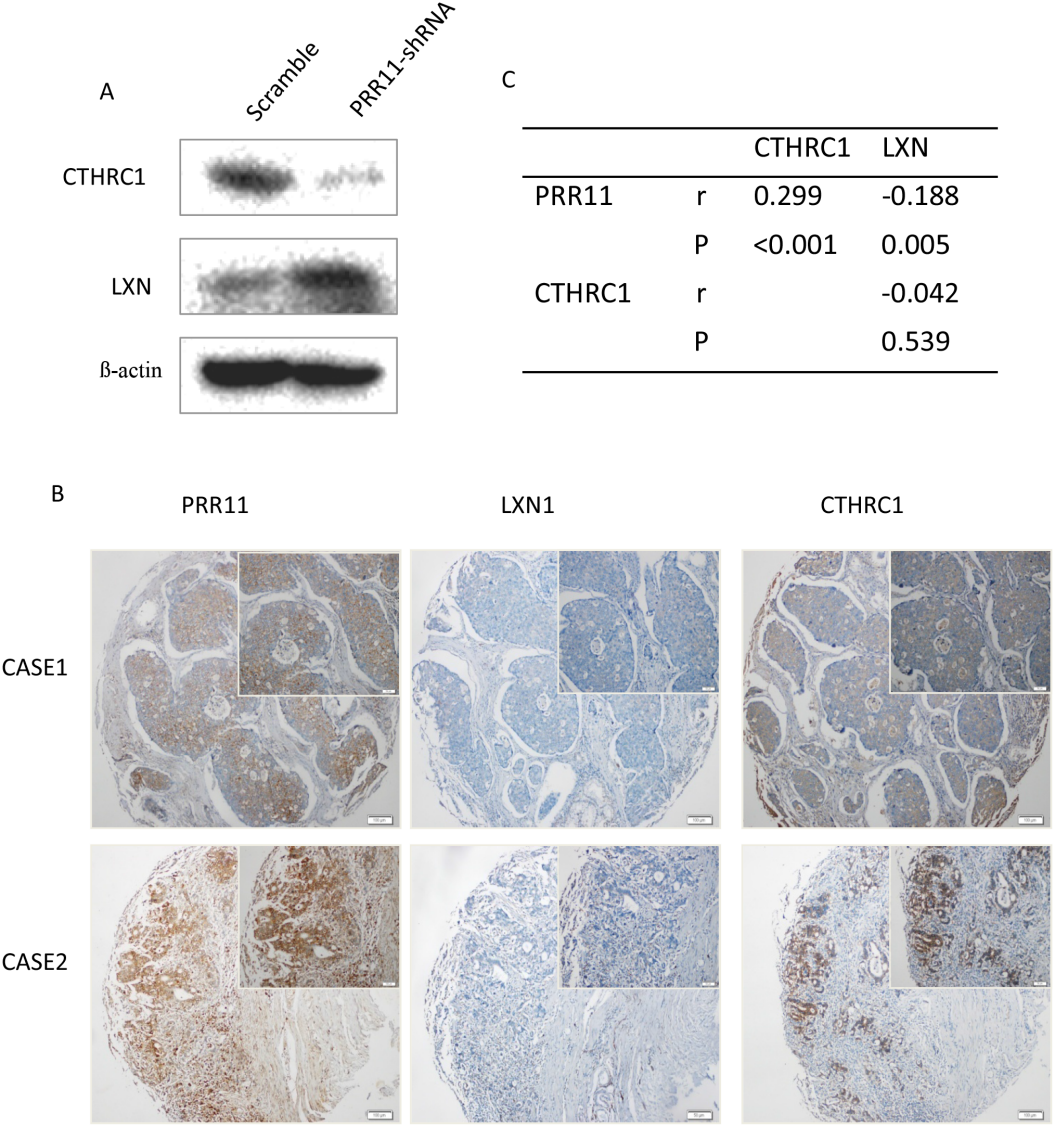
Correlation between expression of PRR11, LXN and CTHRC1 in gastric carcinoma samples. (A) Western blot analysis showing that CTHRC1 is down-regulated and LXN is up-regulated in PRR11-KO SGC07901 cells. (B) Expression of PRR11, CTHRC1 and LXN in patients-derived gastric carcinoma tissue samples. (C) Correlation analysis of PRR11, CTHRC1 and LXN expression. These results were interpreted as showing that CTHRC1 expression is positively associated with PRR11 expression (r = 0.299, p<0.001) and that LXN expression is negatively associated with PRR11 expression (r = -0.188, p = 0.005) in GC tissues.

We performed Western blot analysis to explore whether the expression of CTHRC1 and LXN was altered in PRR11-KO SGC-7901 gastric cancer cells. The results demonstrated that CTHRC1 was downregulated and LXN was upregulated in PRR11-KO cells compared to controls (Fig 4A). Further co-expression analysis was performed in GC tumor samples using immunohistochemistry (Fig 4B). The IHC data confirmed that CTHRC1 expression was positively associated with PRR11 expression (r = 0.299, p<0.001) and LXN expression was negatively associated with PRR11 expression (r = -0.188, p = 0.005) in GC tissues (Fig 4C).

## Discussion

PRR11 is a relatively novel protein molecular that may play a role in cancer pathogenesis, and there is currently only few articles describing the role of PRR11 in lung cancer[13]. While PRR11 was reportedly up-regulated in lung cancer, the possible mechanisms for this increased expression has not been reported, and, moreover, much work is necessary to evaluate the clinical significance of PRR11. In an effort to probe this question, the current study was conducted to characterize PRR11 expression in gastric carcinoma along with any association with clinicopathologic characteristics.

Our study found that both PRR11 mRNA and protein are up-regulated in GC tissues compared with normal mucosa; a finding which supports the notion of a pro-oncogenic role of PRR11 in gastric carcinoma. Moreover, positive expression of PRR11 was significantly associated with an aggressive caner phenotype, including tumors with increased amounts of invasion, increased tumor dedifferentiation, and advanced disease stage. In addition, we have established a gastric carcinoma cell line in which PRR11 is stably knocked down. This cell line demonstrates diminished cellular proliferation and substantially decreased colony formation in our assays. Taken together, these results suggest that PRR11 protein expression may play a critical role in the development and progression of GC cancer.

TNM cancer staging is universally accepted classification system in guiding cancer treatment and predicting prognosis. Unsurprisingly, patients in our study with resections at an earlier stage had a longer survival interval than those with later stage disease. Recently, biological markers have peaked the interest of clinicians as a way to assess/predict therapeutic effectiveness and patient outcomes. Some examples of such markers that are currently in use are HER2, mTOR, and ANXA1 [14–16]. Our study supports a potential prognostic role for measuring PRR11 in gastric carcinoma as Kaplan-Meier analyses demonstrated that positive expression level of PRR11 was associated with poor prognosis in both early TNM stage (TNM I/II) and in late TNM stage (TNM III/IV). Further multivariate analyses revealed that PRR11 overexpression was an independent survival predictor for GC patients after resection of primary tumors. This is the first report that PRR11 expression is associated with poor postoperative outcome in GC patients.

The possible mechanisms underlying PRR11-promoting carcinogenesis have not been fully investigated. The previous study of PRR11 was conducted and interpreted in the setting of lung cancer and showed that PRR11-knockdown dysregulated the expression of multiple important gene in critical pathways such as cell cycle, tumorigenesis and metastasis (e.g. CCNA1, RRM1, MAP4K4 and EPB41L3)[9].

In a proprietary microarray analysis (S1 and S2 Tables)[17], we found that two novel candidate genes (CTHRC1 and LXN) were remarkably altered after PRR11-knocking down. CTHRC1 was first reported as a novel secretory protein in injured and diseased arteries, and it is thought that CTHRC1 may act to inhibit collagen expression and promotes cell migration, both of which are essential functions for an invasive cancer[18]. Further studies demonstrated that CTHRC1 protein is highly expressed in metastatic lesions compared with nonmetastatic tumors, and inhibition of CTHRC1 expression results in decreased cell migration in vitro[19]. Recently, it was shown thatCTHRC1 protein expression is closely associated with the development of liver cancer[20, 21], pancreatic cancer[22], gastric cancer[23], and colorectal cancer[24]. LXN is a relatively novel gene that has been reported to be downregulated in human GC. Canonically, LXN is thought to exhibit tumor suppressor-like functions [25]. It has also reported that LXN has tumor suppressive properties in malignant melanoma[26] and hepatocellular carcinoma [27]. We also showed that overexpression of CTHRC1 was positively correlated with tumor invasion, regional lymph node metastasis, disease progression, and patients’ outcome (S2 Fig), while LXN overexpression was negatively associated with lymph node metastasis, tumor differentiation, and disease stage (S3 Table), indicating oncogenic role of CTHRC1 and suppressive role of LXN in GC. Interestingly, our study demonstrated that silencing PRR11 caused decreased expression of CTHRC1 and increased expression of LXN, indicating a cross-talk between PRR11 and CTHRC1 and LXN. Moreover, there was a significant correlation between PRR11 expression and CTHRC1 and LXN expression in GC specimens. These results suggested that PRR11 might promote cancer progression through the interaction with CTHRC1 and LXN. However, the exact mechanism needs to be further investigated.

In conclusion, PRR11 is frequently expressed in human GC, and its expression is associated with poor survival of GC patients. Additional in vitro and in vivo studies revealed that knockdown of PRR11 inhibits GC cell proliferation, colony forming ability, and tumor growth in a gastric carcinoma cell line. Subsequent correlation analysis revealed that PRR11 expression is also positively correlated with CTHRC1 expression and negatively correlated with LNX expression, providing the hints of a potential mechanism for the oncogenic function of PRR11. The current study provides the groundwork for the future study of these novel prognostic markers which may prove to be useful targets in the detection, screening, and treatment of GC patients.

## Supporting Information

S1 Fig Expression of PRR11 protein in gastric cancer cell lines revealed by Western blotting analysis. (PPTX)

S2 Fig Kaplan-Meier curves of survival durations in patients with gastric cancer according to the expression of CTHRC1 and LXN (A) Patients with CTHRC1 overexpression had a shorter survival duration than those without CTHRC1 expression (53 months vs. 69 months; P = 0.019). (B) No significant difference of overall survival between patients with LXN expression and those without LXN expression (68 months vs. 55 months; P = 0.093).
(PPTX)

S1 Table Down-regulated genes in PRR11-KO cells compared with WT cells in QBC939 cells. (DOCX)

S2 Table Up-regulated genes in PRR11-KO cells compared with WT cells in QBC939 cells. (DOCX)

S3 Table Correlation between CTHRC1 and LXN expression and clinicopathological parameters of gastric cancer (DOC)
