# Supplementary figures and images for: PRR11 Is a Prognostic Marker and Potential Oncogene in Patients with Gastric Cancer
Source: PLoS One. 2015 Aug 7;10(8):e0128943. doi: 10.1371/journal.pone.0128943 (PMC4529228; doi:10.1371/journal.pone.0128943)

## Slide 1
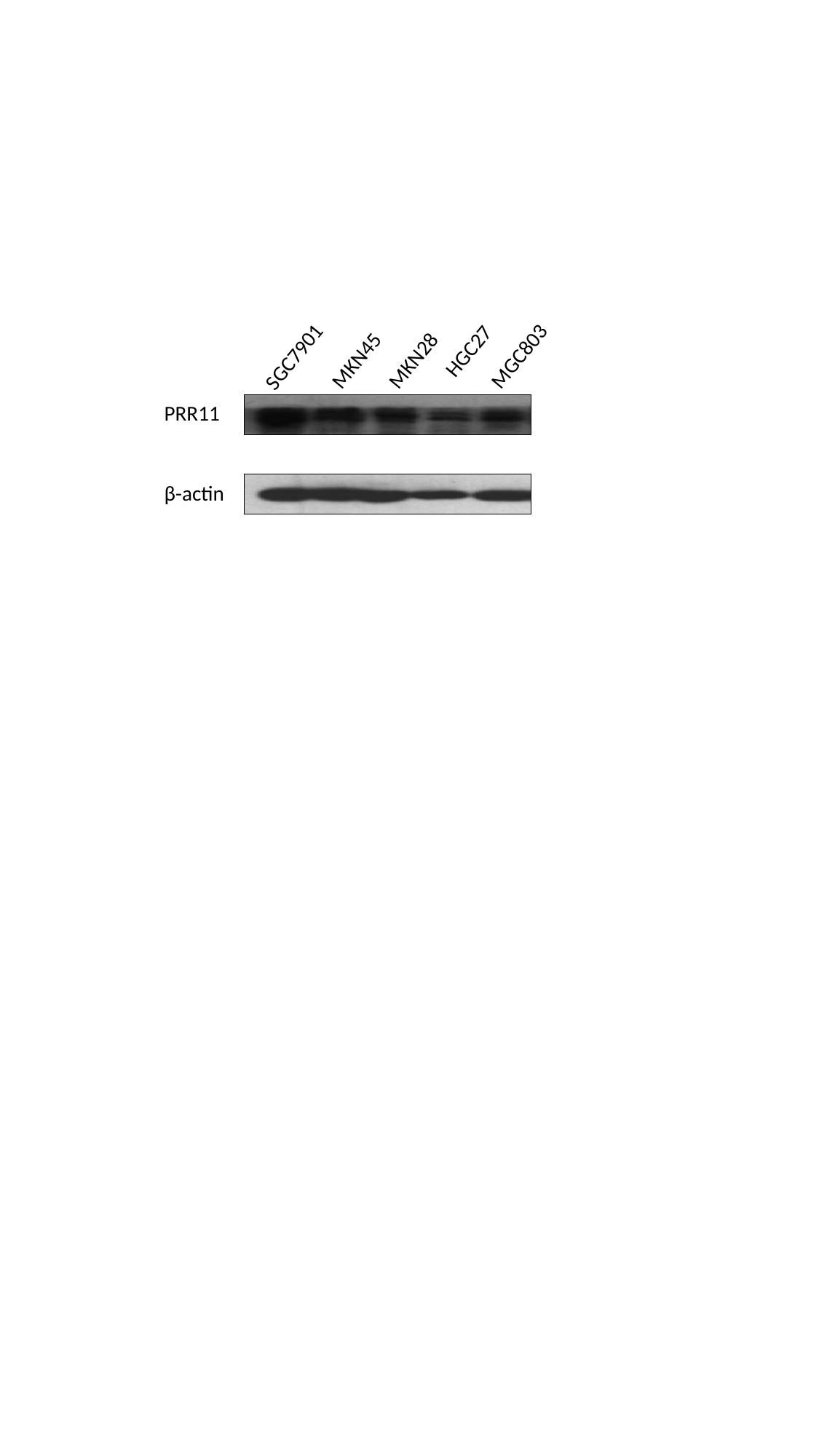

HGC27
MKN45
MKN28
MGC803
SGC7901
PRR11
β-actin

Supplement: S1 Fig — (PPTX) [file pone.0128943.s001.pptx]

## Slide 1
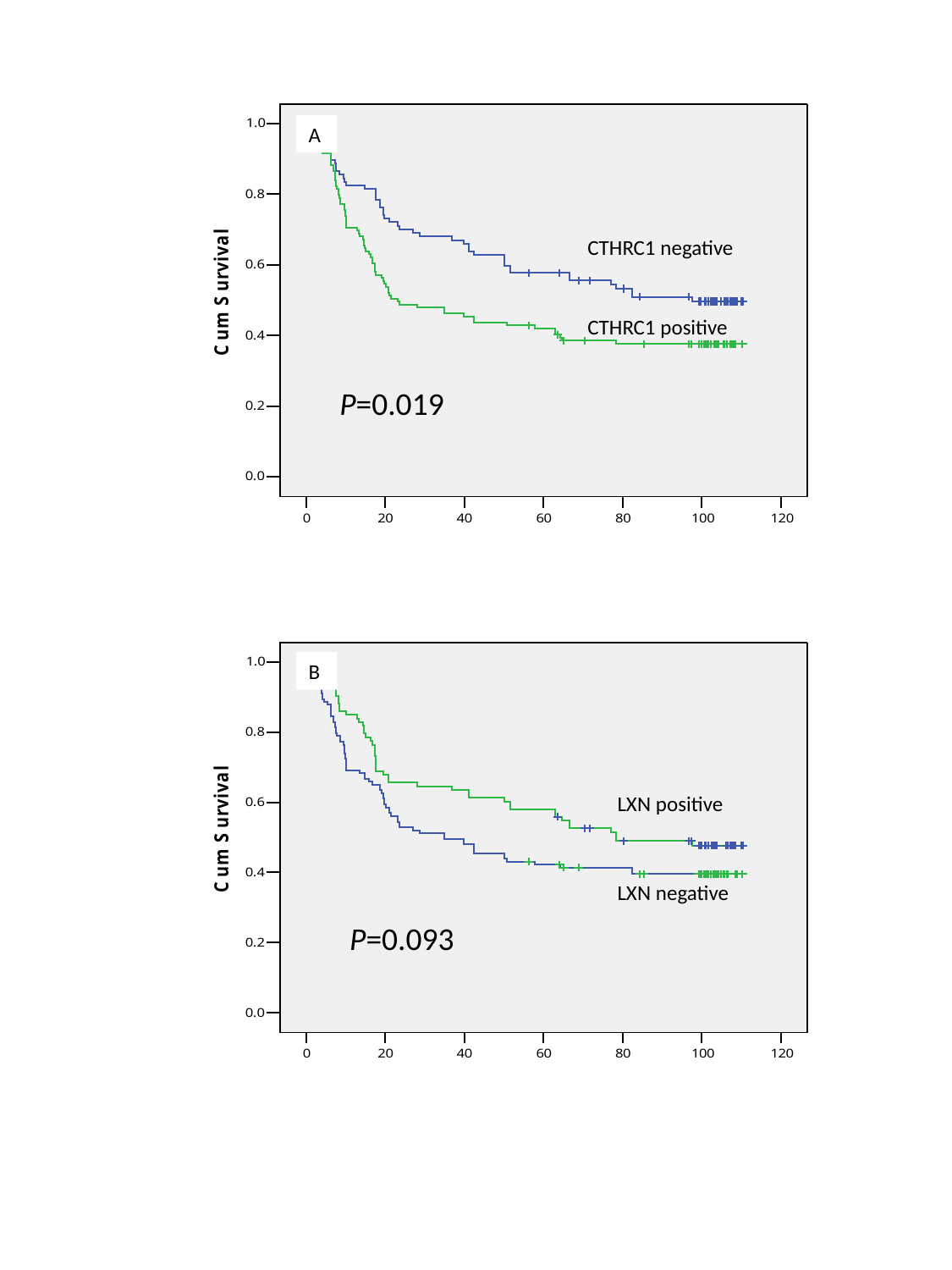

A
CTHRC1 negative
CTHRC1 positive
P=0.019
B
LXN positive
LXN negative
P=0.093

Supplement: S2 Fig — Kaplan-Meier curves of survival durations in patients with gastric cancer according to the expression of CTHRC1 and LXN (A) Patients with CTHRC1 overexpression had a shorter survival duration than those without CTHRC1 expression (53 months vs. 69 months; P = 0.019). (B) No significant difference of overall survival between patients with LXN expression and those without LXN expression (68 months vs. 55 months; P = 0.093). (PPTX) [file pone.0128943.s002.pptx]
