# Supplementary material for: PRR11 Is a Prognostic Marker and Potential Oncogene in Patients with Gastric Cancer
Source: PLoS One. 2015 Aug 7;10(8):e0128943. doi: 10.1371/journal.pone.0128943 (PMC4529228; doi:10.1371/journal.pone.0128943)
Supplement: S1 Table — (DOCX) [file pone.0128943.s003.docx]

Table S1 Down-regulated genes in PRR11-KO cells compared with WT cells in QBC939 cells.

| Fold-change | Regulation ([PRR11-KO] vs [WT]) | Gene Symbol |
| --- | --- | --- |
| 11.2812 | down | UCHL1 |
| 9.874644 | down | DNAH2 |
| 8.381276 | down | ANGPT1 |
| 7.685219 | down | MID1 |
| 7.648831 | down | CTHRC1 |
| 6.390404 | down | SPRR1B |
| 6.083493 | down | TNC |
| 4.644818 | down | AKR1C2 /// LOC100653286 |
| 4.54794 | down | CHCHD5 |
| 4.431042 | down | GPX8 |
| 4.238205 | down | PRR11 |
