# Supplementary material for: PRR11 Is a Prognostic Marker and Potential Oncogene in Patients with Gastric Cancer
Source: PLoS One. 2015 Aug 7;10(8):e0128943. doi: 10.1371/journal.pone.0128943 (PMC4529228; doi:10.1371/journal.pone.0128943)
Supplement: S2 Table — (DOCX) [file pone.0128943.s004.docx]

Table S2 Up-regulated genes in PRR11-KO cells compared with WT cells in QBC939 cells.

| Fold-change | Regulation ([PRR11-KO] vs [WT]) | Gene Symbol |
| --- | --- | --- |
| 15.9254 | up | PYCARD |
| 9.347428 | up | LXN |
| 6.853307 | up | SDR42E1 |
| 6.848017 | up | CEP89 |
| 5.537262 | up | LRRC3 |
| 4.394469 | up | HLA-DMB |
| 3.609359 | up | CAMK1D |
| 3.535861 | up | AKAP12 |
| 3.435173 | up | COL6A1 |
| 3.275157 | up | ARL2BP |
| 3.205708 | up | RAMP1 |
| 3.168427 | up | PSMB9 |
| 3.011879 | up | MTF2 |
