# Supplementary material for: PRR11 Is a Prognostic Marker and Potential Oncogene in Patients with Gastric Cancer
Source: PLoS One. 2015 Aug 7;10(8):e0128943. doi: 10.1371/journal.pone.0128943 (PMC4529228; doi:10.1371/journal.pone.0128943)
Supplement: S3 Table — (DOC) [file pone.0128943.s005.doc]

S3 Table. Correlation between CTHRC1 and LXN expression and clinicopathological parameters of gastric cancer

| Variables | N | CTHRC1 | *P* | LXN | *P* |
| --- | --- | --- | --- | --- | --- |
|  |  | Positive (%) |  | Positive (%) |  |
| Age |  |  |  |  |  |
| ≤60y | 71 | 35(49.3) | 0.231 | 25(25.2) | 0.103 |
| >60y | 145 | 84(57.9) |  | 68(46.9) |  |
| Gender |  |  |  |  |  |
| Male | 149 | 84(56.4) | 0.572 | 64(43.0) | 0.964 |
| Female | 67 | 35(52.2) |  | 29(43.3) |  |
| Tumor size | |  |  |  |  |
| ≤6cm | 180 | 97(53.9) | 0.426 | 80(44.4) | 0.357 |
| >6cm | 36 | 22(61.1) |  | 13(36.1) |  |
| T stage |  |  |  |  |  |
| T1/2 | 78 | 33(42.3) | 0.005 | 36(46.2) | 0.489 |
| T3/4 | 138 | 86(62.3) |  | 57(41.3) |  |
| N stage |  |  |  |  |  |
| N0 | 86 | 36(41.9) | 0.001 | 49(57.0) | 0.001 |
| N1-3 | 130 | 83(63.8) |  | 44(33.8) |  |
| Differentiation | |  |  |  |  |
| Well/moderate | 143 | 77(53.8) | 0.606 | 77(53.8) | <0.001 |
| Poorly/undifferentiated | 73 | 42(57.5) |  | 16(21.9) |  |
| TNM stage | |  |  |  |  |
| I/II | 101 | 45(44.6) | 0.004 | 54(53.5) | 0.004 |
| III/IV | 115 | 74(64.3) |  | 39(33.9) |  |
